# Willingness to pay and willingness to accept in a patient-centered blood pressure control study

**DOI:** 10.1186/s12913-017-2451-5

**Published:** 2017-08-07

**Authors:** Julie Gleason-Comstock, Alicia Streater, Allen Goodman, James Janisse, Aaron Brody, LynnMarie Mango, Rachelle Dawood, Phillip Levy

**Affiliations:** 1 Department of Family Medicine & Public Health Sciences, School of Medicine, Wayne State University, 3939 Woodward Ave, Detroit, MI 48201 USA; 2 Cardiovascular Research Institute, School of Medicine, Wayne State University, 421 E. Canfield St, Detroit, MI 48201 USA; 3 Center for Urban Studies, Wayne State University, 5700 Cass Ave, Detroit, MI 48202 USA; 4 Department of Economics, Wayne State University, 656 W. Kirby St., 2074 FAB, Detroit, MI 48202 USA; 5 Department of Emergency Medicine, Wayne State University, 4201 St. Antoine, UHC-6G, Detroit, MI 48201 USA; 6 Detroit Medical Center, Sinai Grace Hospital, 6071 W. Outer Drive, Detroit, MI 48235 USA; 7 Detroit Medical Center, Detroit Receiving Hospital, 4201 St. Antoine Blvd, Detroit, MI 48201 USA; 8 Integrative Biosciences Center (iBIO), Wayne State University, 6135 Woodward Ave, Detroit, MI 48202 USA

**Keywords:** Hypertension, Patient care and education, Cost evaluation, Survey and questionnaires

## Abstract

**Background:**

Elevated blood pressure is a major risk factor for cardiovascular disease and stroke but patients often discount recommended behavioral changes and prescribed medications. While effective interventions to promote adherence have been developed, cost-effectiveness from the patient’s perspective, has not been well studied. The valuation of patient time and out of pocket expenses should be included while performing cost effectiveness evaluation. The ***Achieve*** **BP** study uses the contingent valuation method to assess willingness to accept (WTA) and willingness to pay (WTP) among patients with a history of uncontrolled blood pressure discharged from an urban emergency department and enrolled in a larger randomized controlled trial.

**Methods:**

WTA and WTP were assessed by asking patients a series of questions about time and travel costs and time value related to their study participation. A survey was conducted during the final study visit with patients to investigate the effectiveness of a kiosk-based educational intervention on blood pressure control. All study patients, regardless of study arm, received the same clinical protocol of commonly prescribed antihypertensive medication and met with research clinicians four times as part of the study procedures.

**Results:**

Thirty-eight patients were offered the opportunity to participate in the cost-effectiveness study and all completed the survey. Statistical comparisons revealed these 38 patients were similar in representation to the entire RCT study population. All 38 (100.0%) were African-American, with an average age of 49.1 years; 55.3% were male, 21.1% were married, 78.9% had a high school or higher education, and 44.7% were working. 55.9% did not have a primary care provider and 50.0% did not have health insurance. Time price linear regression analysis was performed to estimate predictors of WTA and WTP.

**Conclusions:**

WTP and WTA may generate different results, and the elasticities were proportional to the estimated coefficients, with WTP about twice as responsive as WTA. An additional feature for health services research was successful piloting in a clinical setting of a brief patient-centered cost effectiveness survey.

**Trial registration:**

https://clinicaltrials.gov. Registration Number NCT02069015. Registered February 19, 2014 (Retrospectively registered).

**Electronic supplementary material:**

The online version of this article (doi:10.1186/s12913-017-2451-5) contains supplementary material, which is available to authorized users.

## Background

Elevated blood pressure is a major risk factor for cardiovascular disease [1, 2], but patients often discount recommended behavioral changes and prescribed medications. Globally, elevated blood pressure is estimated to cause 12.8% of all deaths and the prevalence of raised/elevated blood pressure in adults aged 25 years and older was around 40% [3]. In the United States, prevalence of hypertension among adults for the interval 2003–2010 was 30.4% (66.9 million) and an estimated 53.5% (35.8 million) of these did not have their blood pressure under control. Non-Hispanic Black adults in an urban environment in the US have the highest prevalence of hypertension (40.4%), as compared to non-Hispanic Whites (27.4%) and Hispanics (26.1%). While effective interventions to promote adherence to prescribed regimens have been developed, the cost-effectiveness of these interventions, particularly from the patients’ perspective, has not been well studied.

The valuation of patient time is an important and often neglected portion of economic evaluation practices in health services research, including cost effectiveness and benefit-cost analysis. Moreover, existing cost effectiveness studies also may not include out of pocket expenses, such as waiting time, childcare, travel, and other transportation costs [4]. Research suggests that even with modest medical expense, travel, time and other socioeconomic issues may limit patient engagement or access to service [5].

Frequently, cost evaluations rely on agency or facility-based data and, because patient-related costs are often not collected or not available, researchers often assign them a value of zero. This omission leads analysts to underestimate costs or to overstate benefit-cost ratios. Realizing this, some health economists seek readily available proxies for time valuation such as minimum wage or average occupational wage. While better than valuing time as zero, this practice does not address individual patient characteristics, implicitly assuming that all people at the same location, or within a specific occupation, place the same dollar value on their time.

A common approach to including patient cost is the contingent valuation method (CVM), which assigns value to a benefit with no market value [6–8]. Typically, CVM is defined as the willing to pay (WTP), which is the maximum amount an individual would be willing to pay to secure a benefit. A related approach is willingness to accept (WTA), which is the minimum amount the individual would be willing to accept to forego the benefit [9]. WTP is commonly used in economics and research has shown its use in environments of universal health care [10] [5] [11]. The use of both WTP and WTA to estimate value is a less common approach, but appropriate for patient-centered research.

The present study demonstrates the use of both WTP and WTA as part of a cost-effectiveness evaluation of an educational intervention, Achieving Blood Pressure Control through Enhanced Discharge (***Achieve*** **BP**). The intervention was developed to increase blood pressure control among patients with a history of uncontrolled blood pressure and seeking care through an urban emergency department.

## Methods

Data for this study were collected within ***Achieve*** **BP**, a larger randomized clinical trial (RCT) which integrated an interactive kiosk with an evidence- based curriculum into the clinical discharge process of an urban emergency department. The specific protocol for ***Achieve*** **BP** is described elsewhere [12]. The primary aim of the RCT was to determine if enhanced discharge from the emergency department using kiosk-based hypertension education modules would improve patient blood pressure control. A secondary aim was to examine the cost effectiveness of the intervention, which was achieved by looking at costs through the numerator of the cost-effectiveness ratio.

The Detroit Medical Center Clinical Research Office authorized the study to take place on May 3, 2013, pending approval of the study protocol by the Wayne State University (WSU) Institutional Review Board (IRB). WSU IRB Full Board Approval (IRB#050213M1F) was received July 23, 2013 and RCT study patient enrollment began in October of 2013. The WSU IRB approved an amendment in June of 2014 for administration of the ***Achieve*** **BP** Cost-Effectiveness Survey.

### Patient enrollment and data collection

The population for the cost-effectiveness study constituted a subgroup of ***Achieve*** **BP** patients who completed the six-month follow-up. The study inclusion criteria were adults (age > 18) without end stage renal disease with a self- reported history of hypertension and who presented with uncontrolled blood pressure (>140/90 for non-diabetics and >130/80 for diabetics) to a large hospital emergency department in Detroit, Michigan for care. Secondary inclusion criteria included English language and the physical and cognitive ability to interact with a kiosk.

All patients, regardless of study arm, received the same antihypertensive protocol, including commonly prescribed medication, assuring standard clinical practice for treatment of blood pressure [13]. Additionally, all patients met with research staff four times over a six-month period and used the same kiosk to answer patient activation and medication adherence study questions.

Because of implementation delay for the cost-effectiveness component, only patients enrolled in AchieveBP after the first quarter (October – December, 2013) with a six-month follow-up visit occurring between June 2014 and May 2015 were included in this study. Thirty-eight (56.7%) of the 67 patients who completed the AchieveBP study protocol had their last visit during the time of the cost-effectiveness study. All of them (18 intervention and 20 control) completed the survey.

### Survey instrument and procedure

The patient survey was adapted from a questionnaire used previously by one of the co-authors to assess personal WTP and WTA among clients attending a methadone treatment facility [9], which in turn was derived in part from the Medical Expenditure Panel Survey (MEPS) [14]. The survey encompassed 14 items asking about travel distance and time to and from the study site, mode of transportation, travel costs, time spent per study visit and four items specific to WTP and WTA. (The survey is shown in Additional file 1.)

Data collection for the cost effectiveness study was conducted during the final study visit, at the University Clinical Research Center, within walking distance from the hospital emergency department. Project staff introduced the survey using a standardized script (Please see Additional file 1). The introduction specified the purpose of the survey and reassured patients that they were not being asked to pay for the project, a misperception voiced by several patients during pilot testing. Staff read each question to the patient and recorded responses. This procedure was illustrative of a patient centered care approach which incorporated consideration of patient health literacy [15–19]. The average completion time was 15 min.

### Data analysis

IBM SPSS Statistics, Version 22.0 was used to analyze the data. Linear regression analysis was performed using WTP and WTA expressed as dollars per hour. Elasticity coefficients were calculated using the formulation (Additional file 2) to relate percentage changes in WTP or WTA to percentage changes in time spent or saved and to evaluate whether increase or decrease in prices would generate different outcomes.

## Results

Patient characteristics for the cost survey group patients and the balance of the ***Achieve*** **BP** patients are shown in Table 1. Slightly more than half of the patients (55.3%) who completed the cost survey were male. Half reported they did not have insurance (50.0%). The majority reported they did not have a primary care provider (55.9%). Most had a high school education or higher (78.9%) and slightly less than half (44.7%) were employed. All (100%) of the patients were African-American/Black. Their average age was 49.1 years, s.d. = 10.567 years), ranging from 21 through 72 years old. Although there were variations in some patient characteristics, (i.e., gender, education) between the two groups, the differences were not statistical significant.

**Table 1:** Patient demographics

Characteristics	Cost-effectiveness (CE) group *N* = 38 (27.3%)	RCT sample^a^ *N* = 101 (72.7%)	*p*-value^b^
Gender			
Male	21 (55.3%)	50 (49.5%)	0.545
Female	17 (44.7%)	51 (50.5%)	
Education			
Less than High School	8 (21.1%)	18 (17.8%)	0.663
High school or higher	30 (78.9%)	83 (82.2%)	
Employment			
Working	17 (44.7%)	50 (49.5%)	0.616
Not working	21 (55.3%)	51 (50.5%)	
Marital Status			
Married	8 (21.1%)	22 (21.8%)	0.926
Not Married	30 (78.9%)	79 (78.2%)	
Primary Care Provide^c^			
Yes	15 (44.1%)	58 (58.6%)	0.144
No	19 (55.9%)	41 (41.4%)	
Insurance Status^d^			
Insured	17 (50%)	63 (67.7%)	0.067
Not Insured	17 (50%)	30 (32.3%)	

^a^Excludes the 38 patients in the cost study sample

^b^ *p-*values were calculated using Pearson Chi-Square test. They are significant at *p* < 0.05

^c^Primary Care Provider was listed as “unknown” for 4 patients in the CE group and 2 patients in the RCT sample

^d^Insurance Status was listed as “Unknown” or “Refused” for 4 patients in the CE group and 8 patients in the RCT sample

### Estimating WTP and WTA with survey data

Two measures of time price were derived from the survey. WTP was assessed as “If it took you *twice* as long as usual to travel to this clinic and you had to pay, what is the MOST money you would be *willing to pay* for each visit?” and “If this clinic were moved right NEXT DOOR to where you live, for your convenience, and if you had to pay, what is the MOST money you would be *willing to pay* for each visit?” Improvement per trip was in minutes. WTA was assessed as “If this clinic were moved back to its original place and offered you money for your inconvenience, what is the LEAST money you would be *willing to receive* for each visit?” Again with deterioration in minutes, the WTA was calculated in $ per hour. For the ***Achieve*** **BP** sample, the mean WTP was $25.78 and WTA was $14.25. See Table 2.

**Table 2:** Mean values of variables used in time price and wage regression

Variables	Mean/$ amount
Travel time (Q2)	36.58 min
Cost for round-trip transportation (Q3)	$6.07
Most money willing to pay for visit (Q7)	$30.56
If took twice as long to travel to clinic and you had to pay, most money willing to pay (Q8)	$25.41
If clinic moved next door, most money willing to pay (Q9) WTP	$25.78
If clinic moved to original place and you were offered $ for inconvenience, what is the least amount willing to receive (Q10) WTA	$14.25

Table 3 displays the regression analyses for WTP and WTA. In calculating time price, setting logs of 0 as equal to 0 (3 observations for WTP and 1 observation for WTA) did not change rankings. Sensitivity analyses that did not drop observations also showed robust findings. Because WTP relates to a 36.58 min *decrease* in travel time, and WTA relates to a 36.58 *increase* in time, the elasticities are proportional to the coefficients. The WTP elasticity is −0.701 and the WTA elasticity is −0.376.

**Table 3:** Regression analyses dependent variable: logs of valuation per hour

Dep Var.	Willingnessto Pay (*N* = 37)	Willingness to Accept(*N* = 37)
	Ln (WTP)	Ln (WTA)
Constant	**4.48339**	**4.05562**
	0.33462	0.22489
Minutes Saved/Spent	**−0.01916**	**−0.01028**
	0.00609	0.00205
Std Error	1.52638	1.02586
R^2^	0.22040	0.41904
Elasticity	−0.70069	−0.37604

Coefficients in **Bold**

## Discussion

Essentially, WTP and WTA are two different ways to estimate the same thing-- the valuation of travel time. Health economists often prefer to use WTP or WTA analysis to determine how much individuals are willing to give up to save designated amounts of time. The value varies by individual. Fifteen minutes of waiting time may be worth $10 to one person, but only $2 to another. These specific values should be factored into evaluations. Just as thirsty people may value the first glass of water much more (higher WTP or WTA) than the second or third, peoples’ WTP or WTA for travel, waiting, or treatment time may depend on the number of minutes of time foregone. For health policy calculations (such as the provision of transportation or the relocation of facilities), impacts would differ depending upon the improvement (or deterioration) of service. Although health economists often use the estimated mean WTP and WTA, health professionals would like to know how these valuations of travel time would vary if accessibility were improved or reduced. Economic theory suggests that incremental willingness to pay is negatively related to time spent or saved, but it is useful to know whether WTP (or WTA) varies “a little” or “a lot” with respect to the time saved. Economists use the term *elasticity* to relate percentage changes in WTP (or WTA) to percentage changes in time spent or saved. The elasticity approach assures that one does not get a different value from measuring value in dollars, euros, or pounds, or time in minutes or hours. This form of standardization is similar to the use of betas in the psychometric literature to relate outcomes of dependent variables to standard deviation changes in explanatory variables [8].

Because time is a cost, reducing patient time is a good thing to do. Negative values of elasticity suggest that a reduction in time will result in lower costs. If the elasticity is −1 or even more negative, we could save some big costs by reducing time spent. If the elasticity is close to −0, we would still reduce costs, but the reduction is not as large because the valuations don’t change very much. Theoretically, small amounts of incremental time are valued most. Further reductions are valued less. If these time valuations are important, they can give guidance for patient activity, e.g., setting up medical clinics within walking distance for patients.

Beyond cost evaluation, estimating the effect of personal costs, particularly time price on client’s treatment attendance, can help to identify and measure barriers to health service and can find ways to diminish or eliminate them. To our knowledge, the ***Achieve*** **BP** study is unique in that primary data collection for both WTP and WTA occurred as the end of the six-month intervention. Two studies, one in Great Britain and the other in Spain, were found that used both the WTA and WTP approach, however, they interviewed patients at the beginning of the clinical process [20, 21]. A strength of this procedure is that the estimations of WTP and WTA are likely based on patients’ cumulative experience with the program, rather than just one event.

## Conclusion

Contingent valuation method studies in healthcare tend to use the WTP approach. However, this study chose to use an adapted health economics model that included WTA, an approach more commonly studied in universal health care environments such as Canada or European countries. As the United States continues to seek provision of affordable healthcare, this approach utilizing WTP and WTA could be of value. An additional feature for potential in health services research was successful piloting in a clinical setting of a brief cost effectiveness survey. Most importantly, the survey is easy to do and can give health planners and medical staff relevant patient-based information for policy formation.

## Additional files

Additional file 1: *Introduction for* ***Achieve*** **BP** *and Patient Cost-Effectiveness Survey*: Administration of the cost-effectiveness survey to patients and survey instrument. (DOCX 25 kb)

Additional file 2: *Elasticity*: Elasticity co-efficients were calculated to relate percentage changes in WTP/WTA to percentage changes in time spent/saved. (DOCX 20 kb)

## Abbreviations

***Achieve*****BP**: Achieving Blood Pressure Control through Enhanced Discharge
CVM: Contingent valuation method
MEPS: Medical Expenditure Panel Survey
RCT: Randomized clinical trial
WTA: Willingness to accept
WTP: Willingness to pay
