# Supplementary material for: Willingness to pay and willingness to accept in a patient-centered blood pressure control study
Source: BMC Health Serv Res. 2017 Aug 7;17:538. doi: 10.1186/s12913-017-2451-5 (PMC5547517; doi:10.1186/s12913-017-2451-5)
Supplement: Supplementary file 1 — Introduction for Achieve BP and Patient Cost-Effectiveness Survey: Administration of the cost-effectiveness survey to patients and survey instrument. (DOCX 25 kb) [file 12913_2017_2451_MOESM1_ESM.docx]

**Additional File 1. *Achieve*BP Introduction and Patient-Centered Cost-Effectiveness Survey**

**Introduction for the *Achieve*BP Patient-Centered Cost-Effectiveness Survey**

*These are the explanations to be used by research staff as they administer questions 1-10 in the cost-effectiveness survey to patients.*

This set of questions is different than the other questions we have asked you before. We are trying to get a sense of how *valuable* this project is to people. Some of the questions we asked earlier were about what you got out of being in the study, but, another way to measure value is in terms of dollars and cents-- a cost/benefit analysis: “are the benefits you get worth the costs of participating?” So, we are saying you are one of the experts helping us understand the benefits of participating in a study that may help more people control their blood pressure.

So, these questions relate to the kinds of costs to you as a participant in the study as well as how much whatever benefits you got from participating are worth to you in dollars. Keep in mind that everything you say is confidential and we will not share your responses with anyone outside the study. We will combine the information from all s to get an average cost and average benefit in dollars and use these averages to determine the cost/benefit of the study.

Please keep in mind that there is no charge at any time to you for being in the study - all project costs have already been paid for by the grant. These questions are only for evaluating the project to see if it is worthwhile to continue it.

Any questions at this point?

In this first part of this questionnaire, I will ask you about how much it cost you to be in the study- we know that you had to make a special trip to come here today so you probably had to pay for transportation- gas or bus fare so these questions are related to that.

(**Ask Questions 1-4**)

**Just before Question 5**:

You probably heard the expression “time is money”; well, the next two questions are related to that idea. We know you could have been doing something else with your time instead of coming here. Maybe you had to take time off work or you could have gone shopping or go out with friends. So spending time with us is a type of cost. So the first question is ….

**Just before Question 6:**

This next question will help us put a dollar value to the time you spent with us ….

**Just before Question 7 and to explain questions 7 – 10. Question 10 concludes the survey.**

This next set of questions may again seem different than the questions you are used to answering for this project, but all together are meant to put a dollar amount to how much you have gotten from participating in the study- the value you received from coming here. These questions ask you to think about how much you would be willing to give to get whatever benefits from the study you received. These are hypothetical (theoretical) questions and are only used to get a relative dollar value of how much the program is worth under different conditions.

***Achieve*BP Patient-Centered Cost-Effectiveness Survey**

ID number: _______ Date: _________ Interviewer__________

We have asked you a lot of questions about how you feel about the study and using the kiosk. This next set of questions will help us understand what costs there are to you in being a participant in this study in terms of the amount of time and money. Your responses will help us determine the value of the study not only in terms of improving health but also in reducing health care costs.

1. How do you usually get here and leave?

Walk/bicycle ___

Drive myself ___

Bus ___

Taxi __

Someone takes me _____

If yes, what is your relationship to that person? ____________________________

Do they have to take time off work to give you a ride? ___Y ___N

1. How much time does it usually take you to travel to the WSU Clinical Research Center/CRC at 275 E. Hancock in Detroit?

___ Hours ___ Minutes

1. About how much does it cost you (or the person who takes you here) for your round-trip transportation?

$ ____________

1. To come here, how much money does it usually cost you for childcare or for care of another person?

$ ____________

1. About how much time do you usually spend at your follow-up meeting? From the time you’ve talked to someone about the study to the time you’ve received your incentive?

___ Hours ___ Minutes

1. Are you employed?

___ Yes → wage rate per hour: $ _______________

(estimate from annual salary divided by number of weeks (minus vacation) x number hours usually work each week)

___No → what is the LOWEST wage per hour it would take for you to accept a job? $ ______

1. If you had to pay for each study visit here, what is the MOST money you would be *willing to pay*?

$ ____________

1. If it took you *twice* as long as usual to travel to this clinic and if you had to pay, what is the MOST money you would be *willing to pay* for each visit?

$ ____________

1. If this clinic were moved right NEXT DOOR to where you live for your convenience, and if you had to pay, what is the MOST money you would be *willing to pay* for each visit?

$ ____________

1. If this clinic were moved back to its original place and offered you money for your inconvenience, what is the LEAST amount of money you would be *willing to receive* for each visit?

$ ____________
