# Supplementary material for: Willingness to pay and willingness to accept in a patient-centered blood pressure control study
Source: BMC Health Serv Res. 2017 Aug 7;17:538. doi: 10.1186/s12913-017-2451-5 (PMC5547517; doi:10.1186/s12913-017-2451-5)
Supplement: Supplementary file 2 — Elasticity: Elasticity co-efficients were calculated to relate percentage changes in WTP/WTA to percentage changes in time spent/saved. (DOCX 20 kb) [file 12913_2017_2451_MOESM2_ESM.docx]

**Additional File 2. Elasticity**

If Willingness to Pay is a function of time traveled T, or W = f (T), then the elasticity of W with respect to T is defined as the percentage change in W per percent change in T.

Mathematically

$$Elasticity=\frac{\left(\%\text{ }change in W\right)}{\left(\% change in T\right)}$$

Our calculation is derived from the regression:

lnW = a + b T.

To get the elasticity formula above, differentiate the equation:

$$\frac{dW}{W}=bdT;$$

Multiplying the right hand side by (T/T = 1),

$$\left(\frac{dW}{W}\right)=b\left(\frac{dT}{T}\right)T$$

Dividing both sides by percentage change in T, *(dT:)/T*

$$Elasticity=\frac{\left(\% change W\right)}{\left(\% change T\right)}=bT$$

This measure means that elasticity is related to the reference value of time. Customarily, we use the mean values of the variables, so here we evaluate at the mean value of T, here 36.58 minutes.

An identical mathematical calculation will derive elasticity with respect to WTA.
